# Development of an Accurate and Robust Air-Coupled Ultrasonic Time-of-Flight Measurement Technique

**DOI:** 10.3390/s22062135

**Published:** 2022-03-09

**Authors:** Benjamin Bühling, Stefan Küttenbaum, Stefan Maack, Christoph Strangfeld

**Affiliations:** Bundesanstalt für Materialforschung und -prüfung, Unter den Eichen 87, 12205 Berlin, Germany; stefan.kuettenbaum@bam.de (S.K.); stefan.maack@bam.de (S.M.); christoph.strangfeld@bam.de (C.S.)

**Keywords:** air-coupled ultrasound, laser Doppler vibrometer, refracto-vibrometry, acousto-optic effect, time-of-flight measurements, in-air ranging, non-destructive testing

## Abstract

Ultrasonic time-of-flight (ToF) measurements enable the non-destructive characterization of material parameters as well as the reconstruction of scatterers inside a specimen. The time-consuming and potentially damaging procedure of applying a liquid couplant between specimen and transducer can be avoided by using air-coupled ultrasound. However, to obtain accurate ToF results, the waveform and travel time of the acoustic signal through the air, which are influenced by the ambient conditions, need to be considered. The placement of microphones as signal receivers is restricted to locations where they do not affect the sound field. This study presents a novel method for in-air ranging and ToF determination that is non-invasive and robust to changing ambient conditions or waveform variations. The in-air travel time was determined by utilizing the azimuthal directivity of a laser Doppler vibrometer operated in refracto-vibrometry (*RV*) mode. The time of entry of the acoustic signal was determined using the autocorrelation of the *RV* signal. The same signal was further used as a reference for determining the ToF through the specimen in transmission mode via cross-correlation. The derived signal processing procedure was verified in experiments on a polyamide specimen. Here, a ranging accuracy of <0.1 mm and a transmission ToF accuracy of 0.3 μs were achieved. Thus, the proposed method enables fast and accurate non-invasive ToF measurements that do not require knowledge about transducer characteristics or ambient conditions.

## 1. Introduction

Ultrasonic time-of-flight (ToF) measurements are a common technique in many research and industrial fields, spanning from ranging applications [1,2,3,4] to human-computer interaction [5,6] to non-destructive testing (NDT) of materials [7,8,9,10,11,12,13,14,15]. The basic concept of ultrasonic ToF measurements is that a signal is transmitted from an ultrasonic transducer and received at a later time by the same or a different transducer. From the time delay between transmitting and receiving the signal, properties such as the speed of sound or the distance travelled can be derived. In ranging applications, the main objective is to localize distant scatterers in front of the transmitter. Thereby, the volume between transmitter and scatterer is filled with a fluid, usually air or water. Knowing the speed of sound of the surrounding fluid, its distance can be calculated from the ToF. This setup is adapted in NDT to investigate solid materials. Instead of a fluid, transmitter and receiver are connected to a test specimen. ToF measurements of the test specimen can be related to the location of defects acting as scatterers [9,10,12] or changes in material parameters via the calculated speed of sound [8,11,14,15]. In many cases, the transducers are coupled directly [8,16,17] or with a couplant [18,19] to the specimen surface to reduce amplitude losses from reflection at the transducer-specimen interface. To speed up the measurements and avoid the use of couplant, the specimens are immersed in liquids [20,21] or air [22,23,24]. Especially in air, the increase in measurement flexibility comes with challenges transmitting sufficient acoustic energy into the air and further into the specimen. These issues are extensively elaborated on in a number of publications [18,23,25].

**Figure 1:**
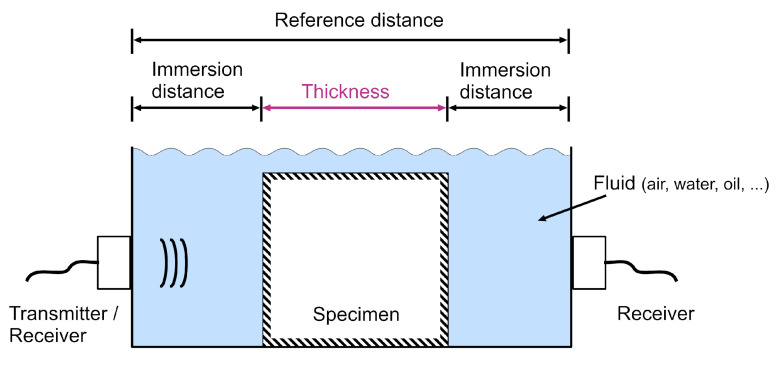
Sketch of a conventional immersed through-transmission setup for ToF measurement. The ToF through the specimen thickness is the target quantity. For an accurate measurement, knowledge of the transmitter characteristics and either the speed of sound in the immersion fluid and the immersion distances or the reference distance are required.

The positioning of the transducer at a distance from the specimen surface further complicates the measurement of the ToF through the specimen, as the travel time of the ultrasonic signal through the immersion fluid amounts to a non-negligible portion of the total ToF. Thus, in addition to determining ToF through the specimen, a measurement needs to be conducted to determine the time delay caused by the immersion. Figure 1 shows such an immersion setup.

Among the methods developed to determine the ToF [2], the correlation approach is considered statistically optimal [26,27] because it uses the entire phase and amplitude information contained in the signal. It requires a reference signal of the immersed transducer to be correlated with the signal received on the opposite side of the specimen. When applied to immersion ultrasound, this method poses a number of challenges. The correlation maximum indicates the time of arrival (ToA) of the signal, which includes the travel time through both the immersion medium and the specimen. The ToF can be determined by subtracting a known time of transmission through the immersion from the ToA. The required reference signal can be modeled if the trigger time of the transducer and its impulse response are known. Since most fluid-coupled ultrasound transducers are triggered electronically, their trigger time can be determined very exactly. However, this is not always the case, as demonstrated by the recently introduced fluidic ultrasonic transducer [28,29]. Although this device is triggered electronically, the sound generation mechanism is governed by fluid turbulences and cannot be controlled precisely, resulting in jitter in the 10–100 μs range. Additionally, if the reference signal is insufficiently modeled, the correlation result gives erroneous information about the actual ToF [30,31]. Direct measurement is then the appropriate method for obtaining an accurate reference signal. If transducers are used that can both transmit and receive, this can be done by recording one or multiple reflections of the transmitted signal. However, this results in a resolution of only half a wavelength [32] and long acquisition times since most immersed transducers are multiple wavelengths away from the specimen. If the transducer does not allow signal sensing, an additional receiver is required close to the transducer. However, this must be secluded from the acoustic axis [21,33], as most sensing devices would interfere with the generated signal. One method to circumvent this challenge is measuring the signal with and without specimen in a transmission arrangement. When measuring without a specimen, the ToA through the reference distance (Figure 1) is obtained. By subtracting the ToA through the specimen from the reference ToA, the ToF can then be calculated [32,34,35]. This differential method requires unchanged environmental conditions, since a change may result in varying waveforms or varying transmission delays through the immersion medium [36,37,38]. In summary, to obtain accurate ToF measurements in a conventional air-coupled ultrasonic transmission setup, the exact acoustic path length and speed of sound in the immersion, the trigger time, and the waveform need to be known.

In this paper, we propose a non-contact method for determining the ToF of a specimen immersed in a fluid that requires no knowledge of these quantities, facilitating measurements in changing environments or using a priori unknown waveforms. This technique is based on refracto-vibrometry (*RV*) using a laser Doppler vibrometer (LDV). *RV* has been previously used for beamforming [39] and qualitative measurements of 2D sound fields [40,41,42]. Tomographic methods have been used to quantitatively reconstruct 3D sound fields [43,44,45,46]. A related acousto-optic approach has been taken by Jia et al. [32] to perform ranging tasks in a water tank. In *RV*, the acousto-optic effect is used to measure sound waves passing perpendicularly through the LDV laser beam. This effect can also be used to provide a suitable non-contact method for receiving an ultrasonic signal close to the specimen surface without influencing the sound field.

In the novel measurement technique introduced in this study, the properties of *RV* sensing are used for accurate non-contact determination of the immersion-induced time delay and to obtain a reference signal for correlation approaches. This procedure allows precise ToF measurements through the specimen for every individual pulse transmitted. Unlike the previously mentioned approaches, the setup presented here does not require a priori knowledge about the exact distance between the transducer and the specimen, the environmental conditions, the signal waveform or the trigger timing and can be performed using commercially available measurement equipment. Additionally, the method allows distance measurements between the laser beam and the specimen when the speed of sound in the immersion fluid is known.

Section 2 presents a brief review of the refracto-vibrometric principle and introduces the theory of the proposed method. In Section 3, the measurement setup used to study the accuracy of this method is presented. In Section 4, the systematic hardware delay is estimated and the measurement results are discussed.

## 2. Theory

The method proposed in this study is based on using the acousto-optic effect to facilitate non-contact ultrasonic time-of-flight measurements. This section briefly reviews refracto-vibrometry, proposes a measurement concept that utilizes its characteristic properties, and explains the relative uncertainty due to misalignment in the setup.

### 2.1. Refracto-Vibrometry

**Figure 2:**
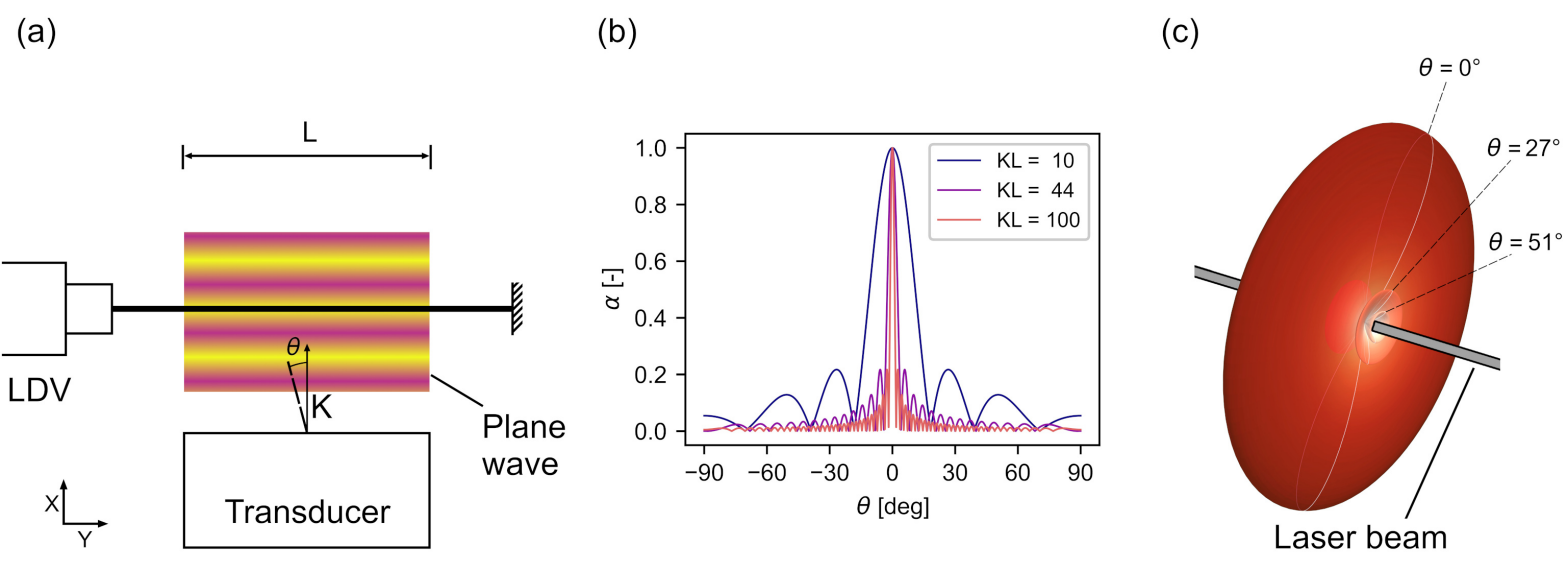
(**a**) Basic refracto-vibrometry setup using a laser Doppler vibrometer. The arrow indicates the propagation direction of the sound waves. (**b**) Directivity of the method for various KL. (**c**) Three-dimensional rendering of the directivity in one point of the laser beam for KL=10.

In *RV*, the LDV laser beam passes through a sound field and is directed at a static reflecting target, as shown in Figure 2. The LDV output signal $s_{RV}\left(t\right)$ is an apparent particle velocity $v_{RV}\left(t\right)$ that corresponds to an integral sound pressure p=p(t,l) along the laser beam. The relationship is given by [44,47]:

(1) $$s_{RV}\left(t\right)=v_{RV}\left(t\right)=\alpha \frac{1}{n_{0}}\left(\frac{\partial n}{\partial p}\right)\frac{d}{dt}\left(\int_{L}p\;dl\right)$$

where α is a directivity factor, *L* is the length of the laser beam intersecting the sound field, (∂n/∂p) is the piezo-optic coefficient, and $n_{0}$ is the refractive index of the immersion fluid. The acoustic field is modeled as a plane wave propagating perpendicular to the laser beam [47] at a sound pressure

(2) $$p\left(t\right)=A\sin\left(\omega t+\varphi_{0}\right)$$

with amplitude *A*, angular frequency ω, and phase $\varphi_{0}$. Inserting Equation (2) into Equation (1), after integration the output signal is obtained as:

(3) $$s_{RV}\left(t\right)=-\alpha \frac{L}{n_{0}}\left(\frac{\partial n}{\partial p}\right)A\omega \cos\left(\omega t+\varphi_{0}\right).$$

Assuming a fixed setup and a sound pressure much smaller than the atmospheric pressure, *L* and (∂n/∂p) are constant [47]. The frequency of the output signal then depends only on the acoustic signal frequency. The amplitude of the output signal depends on the angle of incidence, the acoustic signal frequency, and the amplitude of the sound pressure integrated along the laser beam. It has been shown that the directivity α of a LDV in *RV* mode can be described by a sinc function depending on *L*, the wave number *K*, and the angle of incidence θ [47,48]:

(4) α=|sinc(KLsinθ)|

The resulting directivity for various KL is shown in Figure 2b. As the acoustic frequency or beam width increases, the directivity of *RV* increases. In the case of high-KL non-planar waves, this means that the wave components intersecting the laser beam perpendicularly have the largest influence on $s_{RV}$. However, this directivity only concerns the inclination of the acoustic axis relative to the laser axis. The azimuthal directivity of *RV* for waves passing the laser from different radial directions is uniform, as shown in Figure 2c. Only the inclination θ influences the directivity factor α. The proposed method is based on this property as it allows to capture optically both an acoustic wave generated by a transducer and its reflection by a surface.

### 2.2. Time-of-Flight Measurements

**Figure 3:**
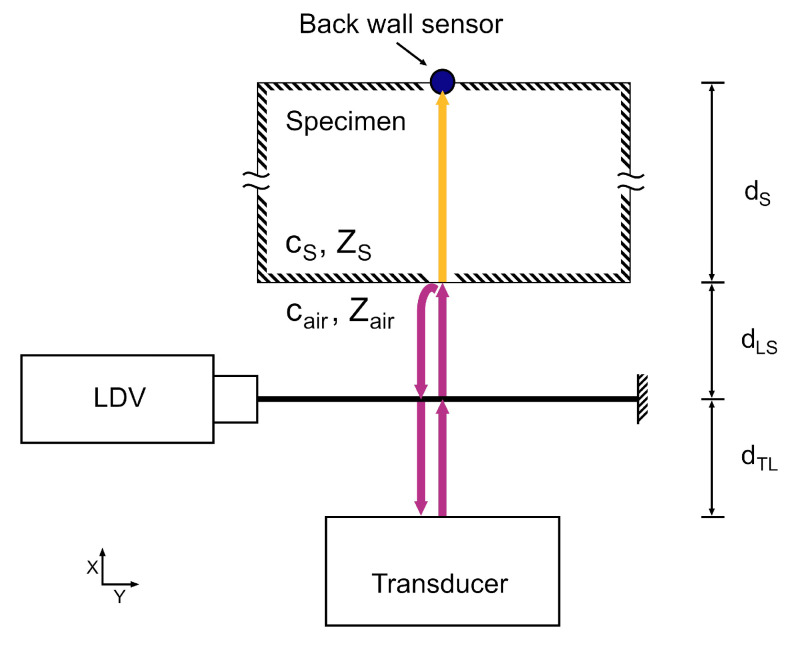
Sketch of the proposed setup. The arrows indicate the sound path through air (purple) and through the specimen (yellow), which are used to determine the signal travel time through air and the specimen. The distances shown are the space between transducer and laser beam ($d_{TL}$), the space between laser beam and specimen ($d_{LS}$), and the thickness of the specimen ($d_{S}$). The sketch additionally shows the speeds of sound ($c_{S}$, $c_{air}$) of specimen and air, causing the time delays, and their specific acoustic impedances ($Z_{S}$, $Z_{air}$), causing the partial reflection of the acoustic signal.

Figure 3 shows a setup that can be used to measure the ToF of an acoustic signal through two media with different specific acoustic impedances *Z*, one of which needs to be optically transparent to the LDV laser beam. This is the case in air-coupled ultrasonic non-destructive testing when the transducer is immersed in ambient air and sends a signal through a specimen. When the acoustic signal is generated by a transducer, it propagates through the air at the acoustic velocity $c_{air}$. It passes the laser beam of the LDV at time

(5) $$\tau_{TL}=d_{TL}/c_{air}$$

where $d_{TL}$ is the distance between transducer and laser beam. As the *RV* is sensitive to all acoustic signals that pass the laser beam perpendicularly, the LDV also records the signals reflected from the specimen surface. The reflected waves pass the laser beam at a time delay of

(6) $$\tau_{2}=\left(d_{TL}+2d_{LS}\right)/c_{air}=\tau_{TL}+2\tau_{LS}$$

where $d_{LS}$ is the distance between the laser beam and the specimen and $\tau_{LS}$ is the time it takes the signal to travel this distance. Knowing $\tau_{LS}$ and $c_{air}$, the distance between the laser beam and the specimen surface can be determined as follows:

(7) $$d_{LS}=\tau_{LS}c_{air}.$$

In NDT measurements, the time delay $\tau_{S}$ through the specimen with thickness $d_{S}$ is the quantity of interest, which is given by:

(8) $$\tau_{S}=d_{S}/c_{S}=\tau_{b}-\left(\tau_{2}-\tau_{LS}\right)$$

where $c_{S}$ is the longitudinal acoustic velocity of the specimen. The signal reaches the back surface of the specimen at $\tau_{b}$, where it is received by a second sensor, such as an additional LDV. The time of reflection $\tau_{LS}$ is exactly the time delay for the wave to couple into the specimen after passing the laser beam. The time of entry into the specimen can be found by autocorrelating $s_{RV}$. The autocorrelation output $R_{11}$ is thus

(9) $$R_{11}\left(\tau \right)=\left(s_{RV}★s_{RV}\right)\left(\tau \right)$$

with ★ being the correlation operator. The secondary peak of the correlation output, ${\hat{R}}_{11}$, is located at the time delay associated with the arrival of the reflected signal, so that

(10) $$2\tau_{LS}=\tau \left({\hat{R}}_{11}\right).$$

The location of ${\hat{R}}_{11}$ can be determined by a suitable peak finding algorithm. The *RV* signal $s_{RV}\left(t\right)$ can be further used to find $\tau_{S}$. This is done by cross-correlating the *RV* signal with signal $s_{2}\left(t\right)$ from a back wall sensor

(11) $$R_{21}\left(\tau \right)=\left(s_{2}★s_{RV}\right)\left(\tau \right).$$

Then the peak correlation output $\tau ({\hat{R}}_{21})$ occurs at $\tau_{LS}+\tau_{S}$. Thus, the ToF can be determined by using Equations (10) and (11) so that

(12) $$\tau_{S}=\tau \left({\hat{R}}_{21}\right)\pm \frac{1}{2}\tau \left({\hat{R}}_{11}\right)-\tau_{h}$$

where $\tau_{h}$ is a delay between the sensors caused by the measurement hardware and the data acquisition system. In case $s_{RV}\left(t\right)$ and $s_{2}\left(t\right)$ represent out-of-phase quantities such as acceleration and velocity, $\tau_{h}$ also includes the resulting phase shift. The sign of the second term of Equation (12) depends on whether the first or the second cross-correlation maximum is chosen, i.e., the cross-correlation result of the back wall signal with the reflected (+) or the incoming (−) in-air pulse.

Consequently, no information about $c_{air}$, $c_{S}$, $d_{TL}$ or $d_{LS}$ are required to determine the ToF $\tau_{S}$. The time signals $s_{RV}\left(t\right)$ and $s_{2}\left(t\right)$ include all the information needed for the calculation via Equation (12). Due to the relational character of the correlation operation, possible jitter in the signal generation and variations in environmental conditions between multiple measurements do not influence the calculated ToF.

### 2.3. Laser Positioning Error

**Figure 4:**
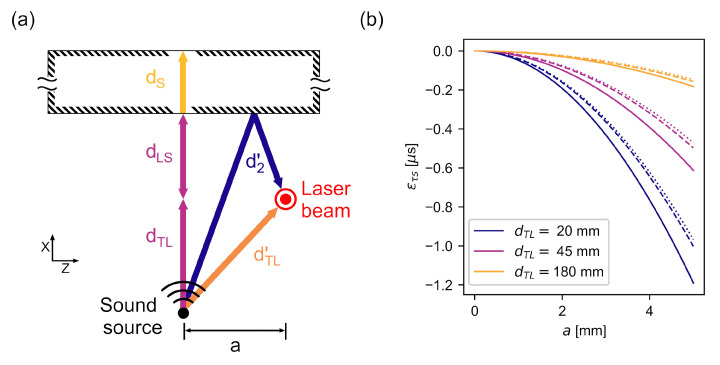
(**a**) Sketch of an erroneous laser positioning. The arrows indicate the sound paths. The coordinate system is rotated about the *x*-axis compared to Figure 3a. (**b**) Calculated ToF due to laser positioning errors for distances that are used in experimental verification. Solid lines: $d_{LS}=20$ mm, dashed lines: $d_{LS}=70$ mm, dotted lines: $d_{LS}=110$ mm.

The theory developed in Section 2 is based on the assumption that the laser beam and acoustic beam axes intersect perfectly. However, errors can occur in the measured ToF if the positioning of the laser beam does not intersect the sound field in its axis. This error is modeled geometrically, which is justified by two assumptions: First, the signal received by the back wall sensor enters the specimen perpendicularly, otherwise it would be refracted off the direct path to the back wall sensor. Second, only the sound waves passing perpendicularly through the laser beam significantly influence the *RV* signal if KL in Equation (4) is sufficiently high. This applies to all wave components in the x-z-plane shown in Figure 4a.

In Figure 4a, the sound paths of the ideal measurement setup are compared with a setup where the laser beam is off the acoustic axis by a distance *a*. The signal has to travel a distance $d_{TL}^{\prime }$ before being sensed by the laser. This distance is given by

(13) $$d_{TL}^{\prime }=\sqrt{d_{TL}^{2}+a^{2}}>d_{TL}$$

Thus, the measured time delay ${\hat{R}}_{21}$ between the signal’s passing through the laser beam and its sensing by the back wall sensor decreases compared to the ideal case as

(14) $$\tau_{r}^{\prime }=\frac{d_{LS}-\left(d_{TL}^{\prime }-d_{TL}\right)}{c_{air}}<\tau_{LS}.$$

On the other hand, the time delay $\tau_{r}^{\prime \prime }$ between the initial and reflected waves passing through the laser beam, calculated using Equation (9), increases since the reflected waves follow a different propagation path $d_{2}^{\prime }$, given by

(15) $$d_{2}^{\prime }=\sqrt{{\left(d_{TL}+2d_{LS}\right)}^{2}+a^{2}}>d_{TL}+2d_{LS}$$

In Equation (12), it is assumed that the time delay $\tau_{LS}$ calculated from Equation (10) represents the wave travel time from the intersection with the laser to the entry into the specimen. However, if the positioning is incorrect, Equation (10) yields the travel time

(16) $$\tau_{r}^{\prime \prime }=\frac{\tau ({\hat{R}}_{11})}{2}=\frac{d_{2}^{\prime }-d_{TL}^{\prime }}{2c_{air}}>\tau_{r}^{\prime }.$$

The resulting time delay error subtracted from the ToF $\tau_{S}$ due to positioning errors can be calculated as

(17) $$\varepsilon_{LS}=\tau_{r}^{\prime }-\tau_{r}^{\prime \prime }=\frac{d_{LS}-\left(d_{TL}^{\prime }-d_{TL}\right)}{2c_{air}}-\frac{d_{2}^{\prime }-d_{TL}^{\prime }}{2c_{air}}.$$

This error has been calculated for various combinations of $d_{TL}$ and $d_{LS}$ in air, which have been investigated in this study and are shown in Figure 4b. The negative error caused by a laser beam *a* deviating from the direct sound path increases strongly as $d_{TL}$ and $d_{LS}$ decrease. The resulting underestimation of $d_{S}$ causes an erroneously reduced $\tau_{S}$ to be calculated from Equation (12).

Using similar geometric considerations, the error from inaccurate positioning of the back wall sensor can be estimated. This error generally increases the calculated $\tau_{S}$ since the path length through the specimen is increased. However, since the longitudinal propagation velocity $c_{S}$ of the specimen is often much larger than $c_{air}$ in NDT applications, this error can be expected to be significantly smaller than $\varepsilon_{LS}$.

The geometrical model used here assumes far-field conditions in which the acoustic wavefronts propagate spherically [49] and are therefore nonparallel to the specimen surface at off-axis locations. A smaller angle between the surface and the wavefront would mean a reduction in $d_{TL}^{\prime }$ and $d_{2}^{\prime }$. The resulting error estimates can therefore be considered as upper bounds.

## 3. Methods and Materials

**Figure 5:**
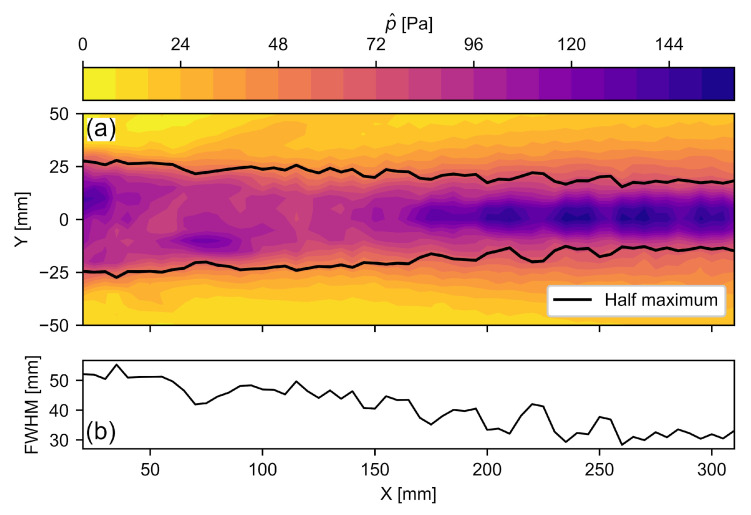
(**a**) Sound field of the Ultran NCG100-S63 transducer in the center plane, where $\hat{p}$ is the maximum sound pressure. The pressure data originate from a previous study [56]. (**b**) The full width at half maximum (FWHM) along the *x*-axis.

To verify the performance of the method, a measurement setup was arranged according to Figure 3. The NCG100-S63 ultrasonic transducer (Ultran Group, Depew, NY, USA) with a center frequency of 80 kHz was used [28]. Its sound field is shown in Figure 5a. Figure 5b shows the beam width *L*, defined by the full width at half maximum (FWHM), which decreases from 50 mm close the transducer surface to 30 mm in the far field. Conservatively assuming L=30 mm for this setup, the resulting aperture of KL=44 causes a high directivity of the *RV* method, as shown in Figure 2. Therefore, mainly the wavefronts with a direction perpendicular to the laser beam and the specimen surface contribute to the *RV* signal. Larger beam widths *L*, as measured close to the transducer surface, result in an even larger directivity. For *RV* sensing, an OFV 3001 LDV (Polytec, Voerde, Germany) was aimed at a fixed aluminum profile at a distance of $L_{0}=1150$ mm from the laser head. The specimen was a polyamide 6 block with dimensions 206.6mm×262.1mm×311.8 mm, and the signal was transmitted through the $d_{S}=206.6$ mm wide dimension. Due to its homogeneity, polyamide is often used as a reference material for bulk wave ultrasonic testing [50,51,52]. A PCB 352M66 accelerometer (PCB Piezotronics, USA) was mounted to the specimen using 1 mm double-sided adhesive tape as the back wall sensor. The signal ToF increase due to the tape is included in the hardware delay $\tau_{h}$ of Equation (12), cf. Section 4.2. A semi-contact setup using an accelerometer was chosen for its high signal-to-noise ratio, which provides a good quality verification of the proposed method [53,54]. The sensor data was recorded with a M2p5966-x4 measurement card (Spectrum Instrumentation, Grosshansdorf, Germany) at 16 bit resolution using a sample rate of 20 MS/s. The recording was triggered at the same instance as the signal generator. To cross-validate the ToF measurement results, ranging measurements were conducted according to Equation (7), using temperature readings from a WS6750 weather station (Techno Line, Dortmund, Germany). Peak finding was conducted by using the find_peaks function of the Python SciPy library [55] and subsequently picking the maximum value of the peaks found.

In refracto-vibrometry, the vibrometer senses the temporal change of the refractive index n(t), so that $s_{RV}\left(t\right)=s_{RV}\left(\partial n/\partial t\right)$. Since n(t) can be considered as a linear function of the acoustic pressure p(t) [44] in the pressure range considered here, it follows that $s_{RV}\left(t\right)=s_{RV}\left(\partial p/\partial t\right)$. Under far-field conditions, this is in phase with the time derivative of the particle velocity ∂u(t)/∂t. The accelerometer measured the particle acceleration at the specimen surface. Thus, $s_{2}=s_{acc}=s_{acc}\left(\partial u/\partial t\right)$ is in phase with $s_{RV}$ and the correlation can be computed according to Equation (12).

## 4. Results and Discussion

Investigating the accuracy of the proposed method requires a detailed look at the signal itself, as acquired by the different sensors, and at all the processing steps needed to compute the result. In this section, after characterizing the signal, the hardware delay between the accelerometer and the LDV is examined. Then, the results of the different geometric parameters $d_{TL}$ and $d_{LS}$ are presented and discussed.

### 4.1. Signal

**Figure 6:**
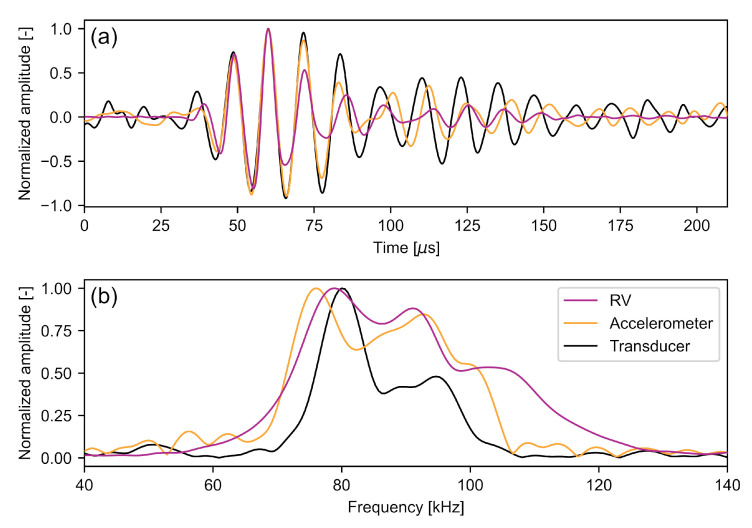
One pulse sensed by the various sensors with $d_{TL}=180$ mm and $d_{LS}=90$ mm in the (**a**) time domain and (**b**) frequency domain. For the signal measured by the ACU transducer, the first reflection was used so that the voltages are not capped. The signals were shifted so that their maxima coincide.

In this study, the ultrasonic signal generated by the ACU transducer is received by various devices: the *RV*-LDV, the accelerometer, and the transducer itself after the signal is reflected by the specimen surface. Figure 6a shows the waveforms of a single signal, measured by all three sensors. All of these signals were band-pass filtered in the range of [20,120] kHz to capture the whole transducer range but filter out high frequency noise, especially in the *RV* signal which has a bandwidth in the MHz range [47].

The acquired waveforms are qualitatively similar having a signal length of about 150 μs. However, slight differences appear more distinct in the frequency domain (Figure 6b). While the maximum frequency peaks of the *RV* and the ACU transducers are very close at 79 kHz and 80 kHz, respectively, the maximum frequency measured with the accelerometer is at 76 kHz. Additionally, a second peak is at 91 kHz for the *RV* data and at 93 kHz for the accelerometer data. These different spectra for the same acoustic pressure burst are caused by the individual frequency responses of the sensors. The piezoelectric transducer itself has a very narrow bandwidth by design, centered at its operating frequency of about 80 kHz. The accelerometer was operated out of its nominal bandwidth, so that the frequency response is not known, but can be assumed not to be flat. The LDV has a sensitivity that is linearly increasing with the frequency, as given in Equation (3). While such differences have little effect in the application of ultrasonic non-destructive testing, they measurably affect the correlation result of Equation (11). The different spectral energy distributions cause a difference in the measured signal period lengths and thus in the envelope shapes, with the latter contributing significantly to the correlation output [29]. This systematic effect is, however, included in the hardware delay of Equation (12) and can thus be corrected.

### 4.2. Hardware Delay

**Figure 7:**
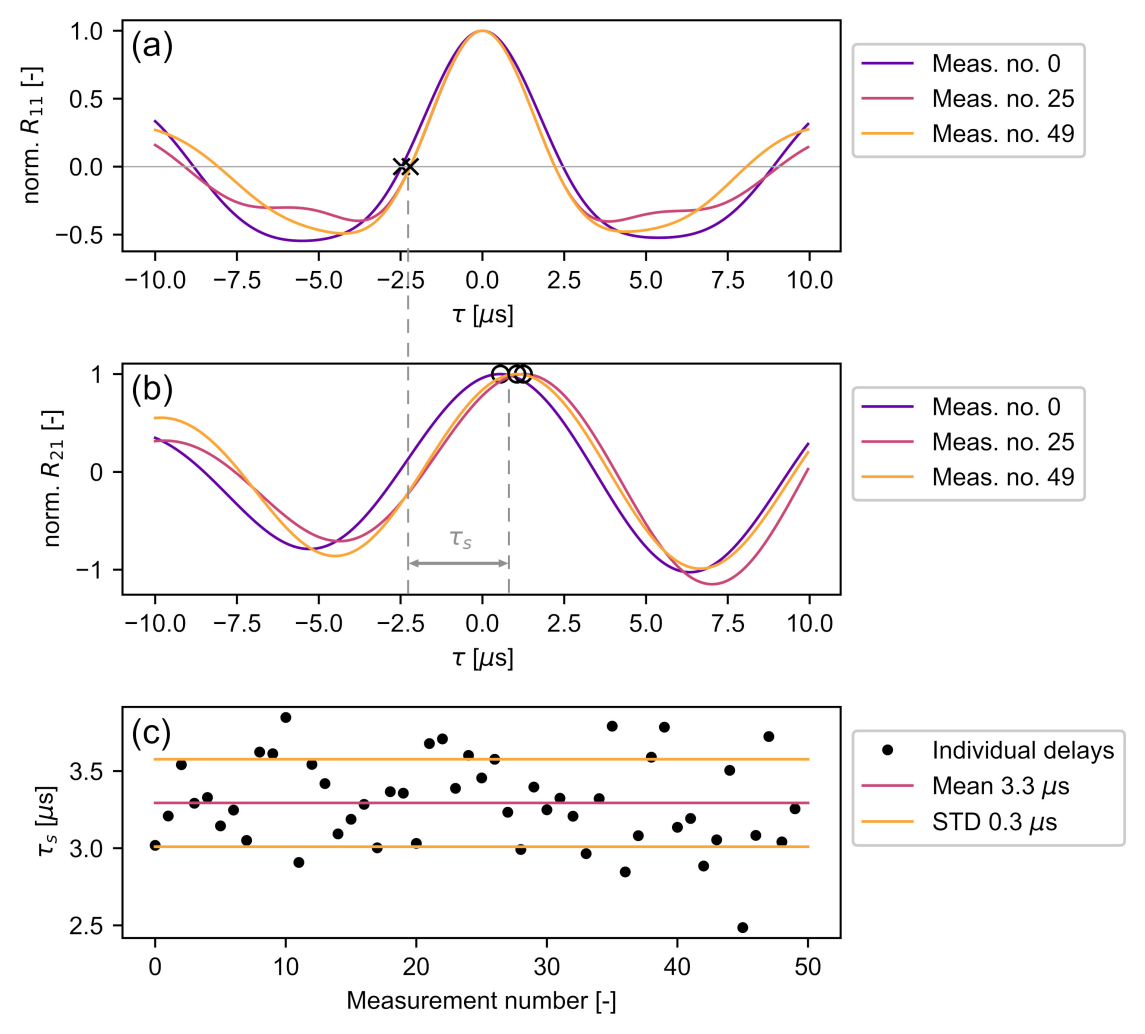
Calculation of hardware delay $\tau_{h}$: (**a**) shows three autocorrelation outputs of the *RV* signal, where × denotes a −π/2 phase shift; (**b**) shows the corresponding cross-correlation outputs of the *RV* and accelerometer data, where ∘ denotes the correlation maximum; (**c**) shows the calculated $\tau_{h}$ for 50 pulses.

The hardware delay $\tau_{h}$ between the sensors was measured by using the LDV in the common vibrometry mode and aiming it at the back surface at a 10 mm distance from the center of the accelerometer. The *RV* signal should be π/2 phase shifted with respect to the accelerometer signal, since the former measures the surface particle velocity and the latter its derivative. If the phase shift deviates from π/2, there is a hardware delay that needs to be corrected. Since the signals are not monochromatic and furthermore have slightly varying frequency content (Section 4.1), it is not sufficient to check only whether the accelerometer signal is −π/2 phase shifted from the peak *RV* signal frequency. All included frequency components need to be considered. The Wiener–Khintchine theorem states that the autocorrelation of a signal is the inverse Fourier transform of its power density spectrum [57]. Thus, the first zero-crossing of τ=0 μs of the autocorrelation $R_{11}$ can be assumed as a good estimate of a mean −π/2 phase shift of the signal, accounting for its mean square spectral content [58]. The autocorrelation output of three individual signals and their zero-crossings indicating a −π/2 phase shift are shown in Figure 7a. Without hardware delay, the cross-correlation maxima of $R_{21}$ (Figure 7b) should be at the location of these zero-crossings. However, the accelerometer signals $s_{acc}$ have a positive delay resulting from varying sensor bandwidth (Section 4.1) as well as internal delays of the sensors, amplifiers, and transmission lines. Figure 7c shows the total hardware delay $\tau_{h}$ of all individual measurements as well as its estimate of 3.3 μs and its uncertainty of 0.3 μs. While the use of a non-contact back wall sensor, such as an additional LDV, is expected to cause a decrease in signal-to-noise ratio, it could provide a reduced hardware delay uncertainty since it provides a flat frequency response over a wide range of frequencies [59].

The LDV measurements were conducted directly on the specimen, while the accelerometer was mounted on the specimen using double-sided adhesive tape. Therefore, this calculated hardware delay includes the additional ToF caused by the tape.

### 4.3. Experimental Verification

**Figure 8:**
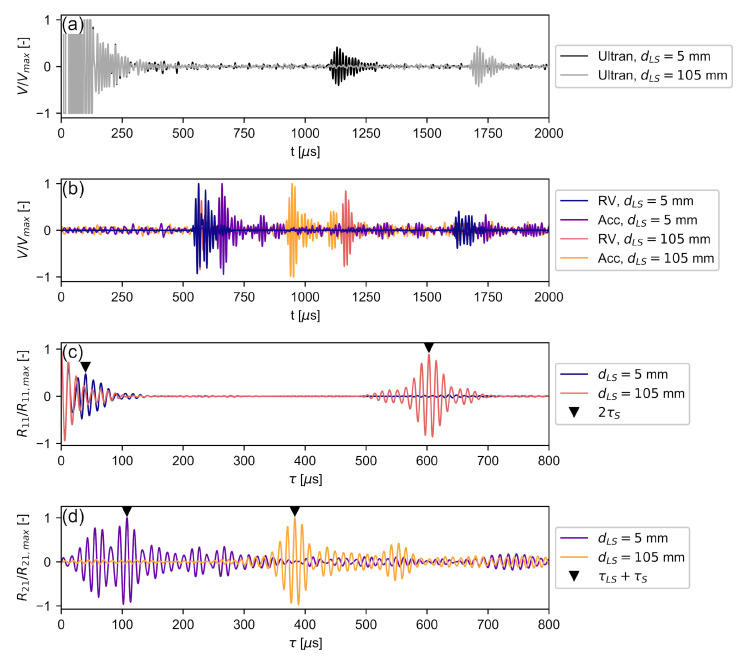
Measurement procedure for $d_{TL}=180$ mm and two different $d_{LS}$: (**a**) shows the time signal from the Ultran piezo transducer as a reference; (**b**) shows the *RV* and accelerometer time signals; (**c**) shows the autocorrelation results of the *RV* signals; (**d**) shows the cross-correlation results of the *RV* and accelerometer signals.

With the hardware delay known, the time delay between the laser and the specimen $\tau_{LS}$ as well as the ToF of the signal through the specimen $\tau_{S}$ were calculated according to Equation (12). This procedure is shown in Figure 8 for two individual measurements with $d_{TL}=180$ mm to highlight a number of characteristic signal and correlation properties. The distances between laser beam and specimen are $d_{LS}=5$ mm and $d_{LS}=105$ mm.

Figure 8a shows the time signals acquired with the Ultran piezoelectric transducer. The voltage of the initial pulse was capped to avoid damaging the data acquisition system. The ringing of the signal lasted to t=250μs, which constitutes the lower time limit for detecting a reflected signal. Its amplitude was previously much lower than that of the driving signal and the subsequent ringing. The first reflections of the pulses were received at t=1100μs and t=1670μs, respectively. The secondary reflections, that would be needed for an accurate determination of the in-air travel time via autocorrelation, are not shown because their late ToA would render the figure illegible. This illustrates the long acquisition time required when the same transducer is used for sending and receiving.

Figure 8b shows the time signals acquired by the LDV in *RV* mode and the accelerometer. Since the distance between the transducer and the laser beam is fixed, the initial waveforms of the *RV* signal match from t=540 μs for both $d_{LS}$. The corresponding accelerometer signals for both configurations followed some time after the initial signal. In the $d_{LS}=5$ mm case, the reflected *RV* signal was received shortly after the incoming pulse, at t=570 μs, due to the small distance between the laser and the specimen. Having a signal length of 150 μs (Section 4.1), the incoming and reflected pulse overlap. Since the time required for the signal to travel back and forth in the air is shorter than to travel through the specimen, the accelerometer signal arrives after the reflected *RV* signal. As the specimen moved away from the laser until $d_{LS}=105$ mm, the reflected pulse was received later in time, starting at t=1140 μs. In this case, the signal that had travelled through the specimen was received in between the in-air signals sensed by *RV*. Since the *RV* receives the in-air pulse much closer to the specimen, a shorter acquisition time is necessary for the autocorrelation of the signal than by using the piezoelectric transducer, although the same signals as in Figure 8a were used. The use of an output signal and a reflected signal that are close in time not only allows for more economical data handling, but also ensures lower attenuation on the propagation path resulting in better signal quality.

Figure 8c shows the autocorrelation results of the *RV* signals obtained from Equation (9). Since the transducer signal is periodic, this autocorrelation result $R_{11}$ does not show a singular peak at $R_{11}\left(\tau =0\right)$, but an envelope with several secondary peaks centered around τ=0 μs. $R_{11}$ also features a secondary envelope caused by the correlation of the incoming and reflected in-air signal. By picking the correlation maximum in this secondary envelope, the two-way travel time of the in-air signal $2\tau_{LS}$ was found. Since the secondary peaks inside the first autocorrelation envelope around τ=0 can be larger than the maximum of the secondary envelope, it was necessary to restrict the peak search to time delays larger than 25 μs, which is approximately two periods of the signal. While the primary and secondary envelopes are clearly separated in the $d_{LS}=105$ mm case, the envelopes overlap in the $d_{LS}=5$ mm case, which is a result of the overlap in the waveform seen in Figure 8b.

Figure 8d shows the results from cross-correlating the *RV* signals with the accelerometer signals following Equation (11). The overlap of initial and reflected waves from the *RV* signal of the $d_{LS}=5$ mm case is visible in the envelope of the corresponding $R_{21}$ in the interval [0,150] μs. The first maximum at τ=55.5 μs represents the cross-correlation of the accelerometer signal with the reflected signal, while the global maximum at τ=107.8 μs represents the cross-correlation with the initial signal, as given by Equation (12). Using the cross-correlation maximum of the initial signal is preferable because the reflected signal may contain interference caused by the signal overlap, which may cause erroneous correlation results. If the in-air pulses sensed by *RV* move further apart, as in the $d_{LS}=105$ mm case, both correlation maxima may be used equally for determining $\tau_{S}$. Since the accelerometer senses the pulse between the initial and received pulse (Figure 8b), the correlation result corresponding to the reflected pulse moves into the negative τ range, which has been omitted in Figure 8d.

The correlation maxima of $R_{21}$ shown in Figure 8d represent the time it took for the signal to travel from its initial encounter with the laser beam at $\tau_{TL}$ to the specimen surface at $\tau_{LS}$, through it ($\tau_{S}$), and be received by the accelerometer with a hardware delay $\tau_{h}$. Since $\tau_{h}$ had been determined earlier, these results were now used to investigate the accuracy of the proposed method when the specimen was moved away from the laser beam. Following Equation (12), $\tau_{LS}$ was determined to calculate $\tau_{S}$ for every pulse.

**Figure 9:**
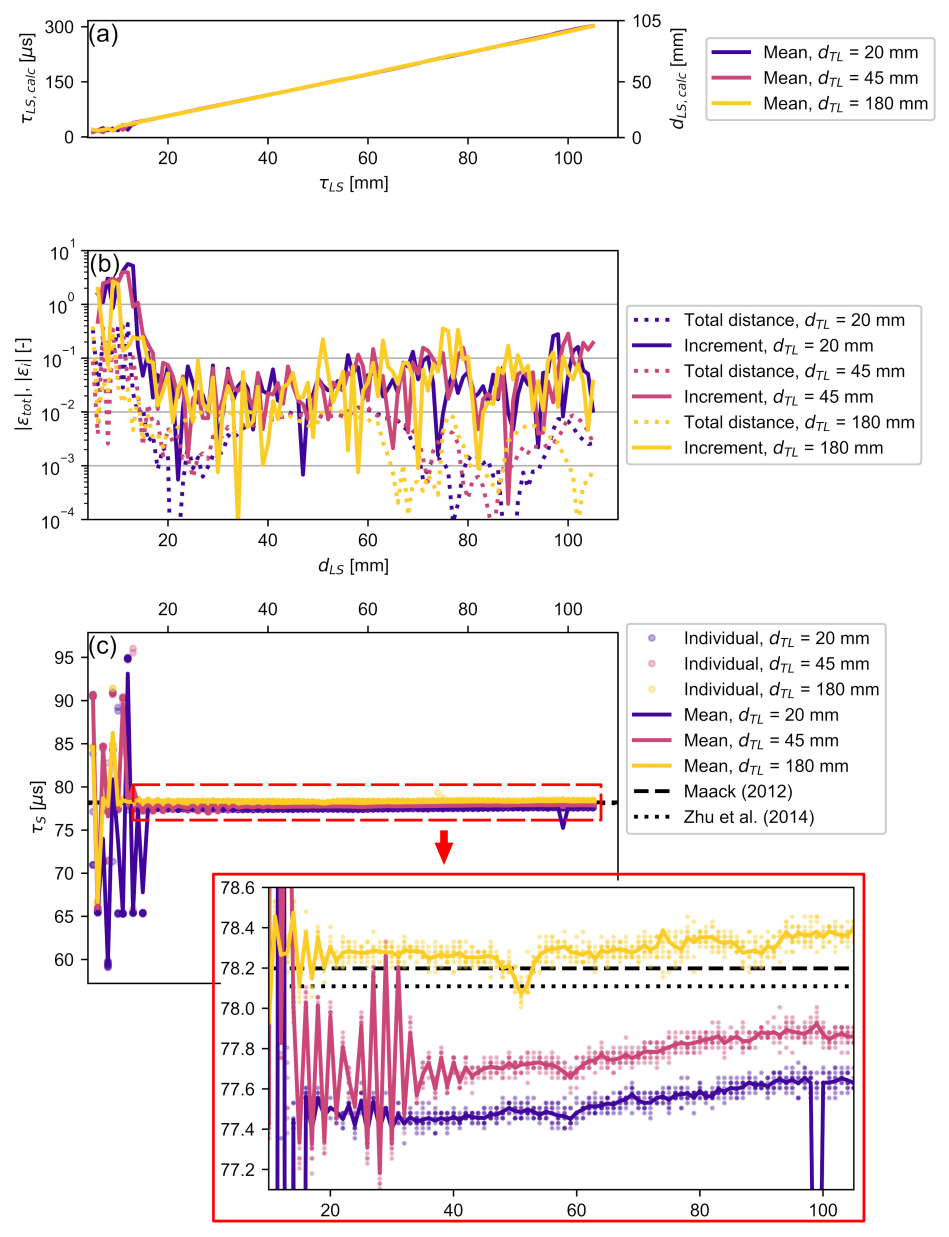
Experimental validation for various $d_{TL}$ and $d_{LS}$: (**a**) shows the mean calculated distance between laser and specimen as well as the corresponding time delay; (**b**) shows the error for this calculated distance for each individual step as well as for the total distance; (**c**) shows the resulting ToF $\tau_{S}$ for each individual pulse and their mean values. Published data [50,60] on longitudinal acoustic velocity in polyamide were used to calculate the reference ToFs. The calculated $\tau_{S}$ for $d_{LS}$ are magnified.

Figure 9a shows the mean calculated time delays $\tau_{LS,calc}$ and distances $d_{LS,calc}$, which qualitatively agree well with the movement positions of the measurement stage. Only for the $d_{LS}<20$ mm cases the results do not follow the set distances linearly. This behavior is caused by the overlap of the autocorrelation envelopes when the laser beam is close to the specimen surface, and by the small distance between the transducer and the specimen, which may cause multiple reflections between the surfaces. Both effects hamper the determination of peak values in the autocorrelation output. Figure 9b shows the absolute relative errors of the total calculated distance $|\varepsilon_{tot}|$ and of each distance increment by which the specimen is moved $|\varepsilon_{i}|$. These are defined as:

(18) $$\varepsilon_{tot}=\left(d_{LS,calc}-d_{LS}\right)/d_{LS}=\left(\tau_{LS,calc}-\tau_{LS}\right)/\tau_{LS}$$

(19) $$\varepsilon_{i}=\left(\Delta d_{LS,calc}-\Delta d_{LS}\right)/\Delta d_{LS}=\left(\Delta \tau_{LS,calc}-\Delta \tau_{LS}\right)/\Delta \tau_{LS}$$

where $\Delta d_{LS}=1$ mm for the measurements presented here. Given the linear relationship between time delay and distance travelled from Equation (7), these errors account for both $\tau_{LS}$ and $d_{LS}$.

For all $d_{TL}$ investigated here, the highest $|\varepsilon_{tot}|=0.4$ is measured when the laser beam is closest to the specimen. This error decreased rapidly to be $|\varepsilon_{tot}|<0.015$ for distances $d_{LS}>14$ mm. The relative total distance error of $d_{LS}=180$ mm dropped at slightly lower distances than the other $d_{LS}$ configurations, which can be attributed to the multiple overlapping reflections between transducer and specimen in the latter cases.

In all three $d_{LS}$ cases, the incremental error $|\varepsilon_{i}|$ reached values >1 in the same region where $|\varepsilon_{tot}|$ was elevated. Except for a small number of outliers (less than 10%), this error dropped below $|\varepsilon_{i}|=0.15$ as $d_{LS}$ increased. A step error of $|\varepsilon_{i}|=0.15$ is equivalent to 3.5% of the signal wavelength. This error per wavelength is of the same order as that reported by Jia et al. [32], who used a comparable method with a higher frequency transducer and water coupling. These results show that *RV* can be used to conduct distance measurements at sub-wavelengths accuracy in air.

As mentioned in Section 2.2, the proposed method neither requires the knowledge of the exact distance between the laser beam and the specimen surface nor the environmental conditions to calculate the ToF through the specimen. Thus, measurement errors in the distance measurements, such as step losses when traversing $d_{TL}$ or in the temperature measurements, are not propagated into the ToF measurements of the specimen. Only the travel times $\tau_{LS}$ were used for determining the ToF in NDT applications. Figure 9c shows the $\tau_{S}$ calculated from Equation (12). They were compared with earlier results by Maack [50], which served as a reference here. The investigated and reference polyamide block were made by the same company. The speed of sound in the reference block was $c_{PA6}=2642$ m/s for signals with a center frequency of 100 kHz. Given the 206.6 mm thickness of the specimen used in this study, the ToF was $\tau_{S}=78.2$ μs. A further value of $\tau_{S}=78.1$ μs using $c_{PA6}=2645$ m/s was included, which was obtained by Zhu et al. [60] using a 2 MHz transducer. Both the maximum parallelism error of the specimen surfaces during production and the thickness measurement accuracy were 0.05 mm, which corresponds to ToF accuracy of 0.02 μs. Thus, the effect of the thickness measurement error on the accuracy of the time delay calculation is smaller than the effect of the sampling interval of 0.05 μs. By far the largest error source is the laser positioning error, which has been estimated to reach up to −1.2 μs for a laser mispositioning of 5 mm.

Similar to the relative distance measurement errors, the calculated ToF deviated considerably when the transducer or the laser beam was close to the specimen surface, i.e., when the initial signal and one or more reflections significantly overlapped. For distances of $d_{LS}>20$ mm, the ToF deviation from the reference values was below 0.8 μs or 1.1%. Following Section 2.3, these deviations imply a laser mispositioning of about 4 mm, which appears excessive considering the effort in assembling the measurement setup and implies that the model employed for the assessment of mispositioning error may need refinement. As the distance between the transducer and laser beam $d_{TL}$ increased, the maximum ToF deviation from the references decreased to 0.4%. This is significantly lower than the thickness measurement error of 1.2% observed by Jia et al. [32] using a differential measurement approach, highlighting the obtainable accuracy of the presented method. Furthermore, a slight increase in ToF with increasing $d_{LS}$ was observed for all configurations investigated. Both behaviors are consistent in amplitude and trend with the errors caused by inaccurate laser positioning (Section 2.3), which decreased with increasing $d_{LS}$. The only outlier from that behavior is found in the $d_{TL}=20$ mm case at $d_{LS}=99$ mm, where the deviation of the individual measurements was about one period of the signal, indicating that a secondary correlation maximum was higher than the correlation value at the true $\tau_{S}$. This is an inherent issue of using correlation methods for undamped narrow-band signals, which are common for piezoelectric ACU transducers. Although the voltage signal-to-noise ratio is high, the correlation peak-to-peak ratio may be marginal (ref. Figure 8d). A higher correlation peak-to-peak ratio may be achieved by generating a Dirac-like acoustic pulse [61] or by using pulse compression techniques [21,22,29,57] that generate a single peak in the correlation output.

## 5. Conclusions

In this study, a novel non-contact method was proposed to provide high-resolution time-of-flight measurements using air-coupled ultrasonic transducers in a transmission setup. The results show how an off-the-shelf laser Doppler vibrometer can be used to obtain accurate ultrasonic time-of-flight measurements. The employed model of the sound paths implies that no prior knowledge is required about the signal waveform, environmental conditions, or even the distance between the transducer and the specimen. The only information needed is the time delay between the sensors used in the setup.

Using a laser Doppler vibrometer, operated in refracto-vibrometry mode as a bidirectional acoustic receiver, the incoming signal and its reflection from a solid specimen surface in-air are sensed. In the first processing step, these data are used to calculated the signal’s time of entrance into the specimen. Then, the same data are used to detect the time-of-flight of the signal through the specimen by cross-correlating it with a signal received on the opposite side of the specimen.

To verify the applicability of the proposed method for different setups, the distance between the laser and the specimen, as well as between the transducer and the laser, were varied using a semi-contact setup with an accelerometer as back wall sensor. It has been shown that a certain minimum distance between the laser and the specimen, here 20 mm, is needed so that the overlap between the direct and reflected signal does not influence the correlation result too much. For the measurement of the time of entrance into the specimen at larger distances, the results show an error per step in the order of 0.4 μs or 3.5% of the signal wavelength when the specimen is moved away from the laser. The overall ranging error of the distance between the laser beam and the specimen is below 1.5%. When calculating the time-of-flight through the specimen itself, the results agree well with the literature and deviate from the reference values by a maximum of 0.8 μs except for a small number of outliers. In the case of the polyamide specimen used in this study, this equates to a maximum offset of 1%. The error approximation due to misalignment of the vibrometer appears to not fully explain this offset. In addition to employing a non-contact back wall sensor, future research should investigate more detailed error models to increase the accuracy of this method even further. Since in this paper it is assumed that some of the inaccuracies are caused by the periodic waveform of the ultrasonic pulse, the accuracy obtained by using coded waveforms is expected to be even higher and should be investigated in the future alongside applications in other fluids.

## Data Availability

The data presented in this study are available on request from the corresponding author.
